# Commentary: Epitranscriptomic regulation by m6A in immunity and autoimmune disorders: emerging mechanisms and clinical perspectives

**DOI:** 10.3389/fimmu.2026.1930681

**Published:** 2026-09-03

**Authors:** Shengchao Zhang, Tong Li, Yong Liu

**Affiliations:** 1 Department of Rehabilitation Medicine, the First Affiliated Hospital of Dalian Medical University, Dalian, China; 2 College of Health-Preservation and Wellness, Dalian Medical University, Dalian, China

**Keywords:** autoimmune diseases, context dependence, functional regulatory modules, m6A regulators, N6-methyladenosine (m 6 A)

## 1 Introduction

We read with great interest the recent article by Maqsood and colleagues titled “Epitranscriptomic regulation by m6A in immunity and autoimmune disorders: emerging mechanisms and clinical perspectives”. Maqsood et al. (1) systematically reviewed the role of N6-methyladenosine (m6A) RNA methylation in immune regulation and autoimmune diseases, with particular emphasis on the functions of writers, erasers, and readers across immune-cell types and disease contexts. A major strength of their review is that it does not portray m6A as uniformly pro- or anti-inflammatory, but instead highlights that its effects are shaped by cell type, disease stage, tissue microenvironment, and inflammatory status.

However, the central limitation is not the absence of context dependence, but the lack of an operational, multiaxial framework for defining and testing how cell lineage, cell state, disease stage, tissue niche, and inflammatory cues interact. Although the review provides a useful synthesis across immune cells, disease categories, and therapeutic strategies, it does not fully define how these variables and their interactions should be controlled, compared, and modeled, or what level of experimental evidence is required to distinguish bona fide m6A-dependent mechanisms from correlative changes in regulator expression. Future studies should therefore move beyond merely acknowledging context dependence and instead establish testable, context-resolved frameworks for mechanistic validation and clinical translation.

## 2 From regulator expression to functional modules

A major risk in current studies of m6A in autoimmune diseases is the overinterpretation of regulator expression. Altered expression of METTL3/METTL14, FTO/ALKBH5, or YTHDF/IGF2BP family members is often used to infer changes in m6A deposition, demethylation, or RNA stability, translation, and decay (1, 2). Such observations are useful for hypothesis generation but are insufficient to establish mechanism. Moreover, differential m6A peaks identified by enrichment-based profiling can show limited reproducibility, further supporting the need for orthogonal validation (3). Changes in a single regulator do not identify the relevant target transcript, reader protein, RNA fate, or downstream immune effect; therefore, assigning a fixed pro- or anti-inflammatory role to a given regulator may lead to mechanistic misclassification.

The evidence summarized by Maqsood et al. (1) also argues against a regulator-centered interpretation. In dendritic cells, METTL3 loss impairs DC activation and CD4+ T-cell priming (4). In CD4+ T cells, METTL3 expression changes with activation and effector differentiation, while METTL3 inhibition alters T-cell activation and Treg differentiation through m6A-dependent regulation of Foxp3 mRNA (5). METTL3 can also regulate T-cell homeostasis through the SOCS/STAT5 axis and promote Tfh differentiation by stabilizing TCF7 mRNA (1, 6, 7), illustrating functional-state-dependent outputs of the same writer.

**Figure 1:**
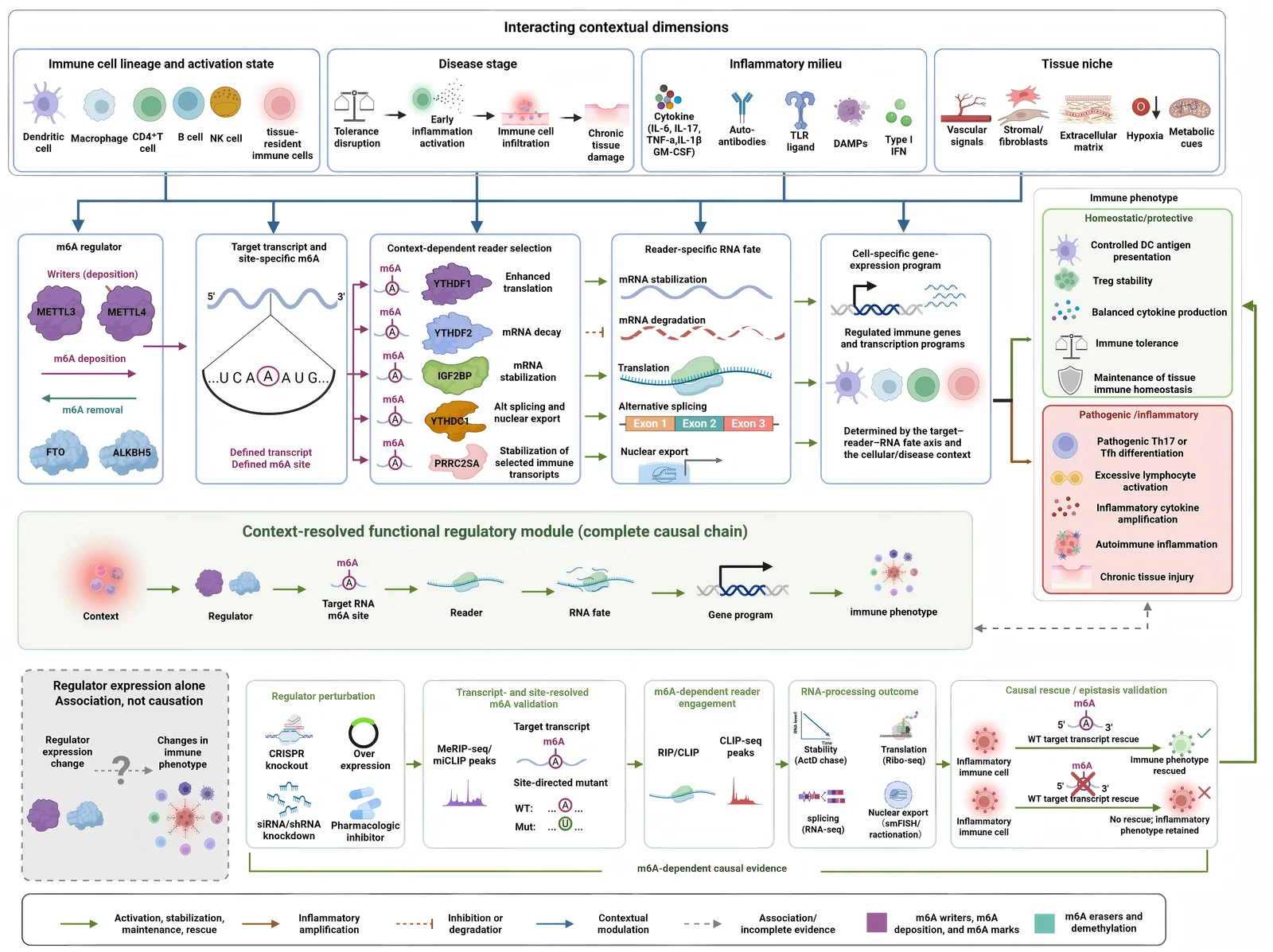
Context-resolved functional regulatory module and causal-evidence framework for m6A-mediated immune regulation. Interacting dimensions—including immune-cell lineage and activation state, disease stage, inflammatory milieu, and tissue niche—shape the operation of m6A regulatory modules. Writers and erasers regulate m6A deposition or removal at defined sites on target transcripts, whereas context-dependent reader engagement influences RNA stability, degradation, translation, alternative splicing, or nuclear export. These RNA-processing outcomes contribute to cell-specific gene-expression programs and homeostatic/protective or pathogenic/inflammatory immune phenotypes. The central sequence summarizes the proposed module as context–regulator–target RNA/m6A site–reader–RNA fate–gene program–immune phenotype. Regulator-expression changes alone indicate association rather than causation. The lower workflow illustrates the proposed evidence required to support an m6A-dependent mechanism: regulator perturbation, transcript- and site-resolved m6A validation, assessment of reader engagement, RNA-processing assays, and rescue validation using a wild-type target transcript and an m6A-site mutant control. Green arrows indicate activation, stabilization, maintenance, or rescue; brown arrows indicate inflammatory amplification; dotted inhibitory lines indicate inhibition or degradation; blue arrows indicate contextual modulation; and gray dashed arrows indicate association or incomplete evidence. Purple denotes m6A writers, deposition, and m6A marks; teal denotes m6A erasers and demethylation.

Accordingly, the basic interpretive unit in m6A mechanistic studies should not be an isolated regulator, but a context-resolved functional regulatory module comprising disease stage, tissue niche, immune-cell state, m6A regulator, target transcript and m6A site, reader protein, RNA fate, gene-expression program, and immune outcome (**Figure 1**). Future studies should establish a minimum causal standard for m6A-dependent mechanisms by demonstrating regulator perturbation, altered m6A sites on specific transcripts, changed reader binding, modified RNA stability/splicing/export/translation, and rescue of the immune phenotype through restoration of the target transcript, reader interaction, or m6A site, as exemplified by mechanistic dissection of the METTL3–Tcf7 axis (7). Without this evidence chain, changes in regulator expression are better interpreted as regulatory associations rather than definitive m6A-dependent causal mechanisms.

## 3 Operationalizing context dependence through a conditional multiaxial framework

Interpreting contradictory findings as context-dependent is straightforward; the greater challenge is to incorporate contextual variables prospectively into experimental design rather than invoke them retrospectively. These variables should not be arranged into a fixed hierarchy, but treated as interacting dimensions operating across distinct biological scales. Their relative contributions may therefore vary across disease phases and depend on interactions between dimensions.

### 3.1 Defining contextual dominance

For analysis, cell lineage and subset should be resolved first to minimize cell-composition confounding and define the potential response space. This analytical precedence does not imply biological dominance. Lineage-associated competence is illustrated by the differential requirement for METTL3 among innate lymphoid-cell subsets: METTL3 deficiency markedly impairs activated ILC2 responses through destabilization of *Gata3* mRNA, while having limited effects on cytokine-induced ILC1 and ILC3 responses (8). Cell state is likewise relevant: T-cell-specific ALKBH5 loss reduces pathogenic CD4+ T-cell responses in experimental autoimmunity through m6A-dependent regulation of Ifng and Cxcl2 transcripts (9). Disease stage should not be considered subordinate to cell state because they operate at different scales. In type 1 diabetes, β-cell METTL3 levels increase at disease onset but decline during progression, demonstrating stage-dependent m6A regulation (10). Tissue niches may further reshape m6A-dependent immune outputs. In rheumatoid arthritis, hypoxia increases ALKBH5 expression in fibroblast-like synoviocytes and promotes inflammatory and aggressive phenotypes through m6A-dependent regulation of CH25H mRNA (11). Spatial studies in rheumatoid arthritis, multiple sclerosis, and psoriasis likewise identify disease-, lesion-state-, or severity-associated tissue niches (12–14), although they did not directly examine m6A regulation.

Accordingly, contextual dominance should be defined within a specified cell population and disease window. A dominant driver may be an individual factor or interaction whose contribution remains reproducible after accounting for other dimensions and whose causal role is supported by single- or combined-factor perturbation that alters the relevant m^6^A module and phenotype.

### 3.2 Experimental identification of context-dependent drivers

Whether niche signals override lineage-associated competence, or inflammatory cytokines become dominant during defined temporal windows, should be tested rather than assumed. Future studies should resolve lineage and subset identity, then compare the same lineage across cell states, disease stages, tissue niches, and inflammatory exposures, testing both main and interaction effects.

Such comparisons should preferably use matched factorial or focused crossed designs rather than combine observations from unrelated cell types or disease models. The same population and regulator–target RNA–reader module should be examined across selected contextual conditions, allowing individual effects to be distinguished from context-dependent interactions.

Time should also be incorporated explicitly. Endpoint measurements cannot distinguish a factor involved in disease initiation from one that maintains, or merely reflects, established inflammation. Longitudinal sampling and perturbation at defined disease windows can address this distinction. Perturb-seq provides a precedent for high-dimensional perturbational dissection of regulatory circuits in immune-response systems (15), whereas inducible m^6^A perturbation may allow the same pathway to be tested across disease stages (16, 17).

Operationalizing this framework requires complementary assays. Single-cell m^6^A approaches, including scDART-seq and scm^6^A-seq, can reveal cell-to-cell m^6^A heterogeneity (18, 19). Long-read nanopore RNA sequencing enables isoform-resolved epitranscriptomic analysis (20), while miCLIP2, reader-specific CLIP approaches, multiplexed m^6^A-seq2, and m6Anet provide complementary site-, reader-, and modification-level information (21–24). These methods should be combined in matched samples with perturbation and rescue experiments, because no single platform captures the entire causal chain.

For tissue-niche effects, spatially resolved perturbation may be particularly useful because it preserves local cellular relationships and can help distinguish cell-intrinsic from microenvironment-dependent effects (25).

### 3.3 From association to causal dominance

The relative contributions of contextual variables and their interactions should be quantified rather than inferred from differential expression alone. Factorial or multivariable models can explicitly estimate main and interaction effects, while mixed-effects and variance-partitioning approaches can quantify the relative contribution of contextual dimensions to variation in m^6^A regulation and immune phenotypes (26, 27).

Statistical predominance does not establish causality. A candidate factor or interaction should remain robust after accounting for other dimensions and be validated by single- or combined-factor perturbation that alters the predicted m^6^A pathway and phenotype, with site-specific or inducible m^6^A editing and rescue providing further validation (16, 17).

Thus, the relevant question is not which contextual dimension is universally dominant, but which factor—or interaction between factors—has causal priority within a defined cell population and disease window. This distinction converts context dependence from a retrospective explanation into a testable framework.

## 4 Discussion

Overall, Maqsood et al. (1) provide a valuable synthesis of m6A regulation in autoimmune disease. However, the field must move beyond regulator-expression changes toward context-resolved causal modules linking regulators, modified transcripts, readers, RNA fate, and immune phenotypes.

Key questions remain. Rather than seeking a universally dominant contextual variable, future studies should determine which factor—or interaction among lineage, cell state, disease stage, tissue niche, and inflammatory cues—has causal priority within a defined cell population and disease window. This requires matched and longitudinal designs that distinguish independent from interaction effects and test whether perturbation of the candidate determinant alters the predicted m6A pathway and phenotype. Importantly, the absence of a single dominant factor should not be considered inconclusive if reproducible interactions better explain the disease trajectory.

Can computational models integrating single-cell, spatial, long-read, CLIP-seq, and quantitative m6A data predict context-specific regulator–target RNA–reader modules? Can context-dependent m6A signatures be validated as biomarkers for disease stage, activity, flare, tissue damage, or treatment response? Can m6A manipulation be restricted to specific cells, tissues, or transcripts without disrupting immune homeostasis elsewhere? These questions are particularly relevant to the translation of context-resolved m6A mechanisms into biomarkers and therapeutic targets.

Answering these questions will require longitudinal, multi-tissue sampling, factorial perturbation–rescue designs, orthogonal m6A mapping, quantitative assessment of main and interaction effects, external cohort validation, and cell-selective delivery. Converting context-dependence from a descriptive concept into a testable and ultimately predictive framework is essential for reliable biomarker development and precision therapy in autoimmune disease.
